# Supplementary material for: Symptoms associated with a COVID-19 infection among a non-hospitalized cohort in Vienna
Source: Wien Klin Wochenschr. 2022 Apr 13;134(9-10):344–50. doi: 10.1007/s00508-022-02028-9 (PMC9007045; doi:10.1007/s00508-022-02028-9)
Supplement: Supplementary file 1 — Supplementary data and analyses [file 508_2022_2028_MOESM1_ESM.pdf]

Supplementary Table 1: Users characteristics

	total	C19+	C19-	Unclear
Total	9133	2692	6341	100
0-16	711	198	508	5
16-20	618	187	425	6
21-30	3142	850	2261	31
31-40	2365	659	1685	21
41-50	1068	333	720	15
51-60	775	271	492	12
61-70	285	115	164	6
71+	151	73	75	3
Age unknown	18	6	11	1
Female	4986	1398	3538	50
Male	4093	1279	2766	48
Intersex	2	2	0	0
Sex unknown	52	13	37	2
Fatigue	6514	1988	4456	70
Hyposmia	1375	701	648	26
Sore Throat	4706	1287	3373	46
Cough	4347	1610	2686	51
Dyspnea	1448	404	1027	17
Rhinorrhea	3596	1027	2532	37
Malaise	6890	2117	4693	80
Dysgeusia	1713	777	910	26
Sneezing	3273	913	2329	31
Fever	3839	1339	2446	54
Diarrhea	1421	317	1086	18
Headache	5229	1714	3457	58
COVID-19 Contact	2449	1096	1324	29

Supplementary Table 2: P-values, test statistics, corrected P-values, symptom combination frequencies, expected symptom combination frequencies for all three-wise combinations of symptoms within C19+

Symptom 1	Symptom 2	Symptom 3	P-value	Test statistic	Corrected P-value	Symptom combination frequency	Expected symptom combination frequency
Hyposmia	Sore_Throat	Fever	4.41E-05	1.67E+01	4.20E-03	5.72E-02	6.19E-02
Hyposmia	Rhinorrhea	Malaise	3.87E-05	1.69E+01	4.20E-03	9.73E-02	7.81E-02
Sore_Throat	Dysgeusia	Headache	1.88E-05	1.83E+01	4.20E-03	1.14E-01	8.79E-02
Hyposmia	Sneezing	Fever	8.68E-05	1.54E+01	4.44E-03	4.75E-02	4.39E-02
Fatigue	Cough	Dysgeusia	1.02E-04	1.51E+01	4.44E-03	1.57E-01	1.27E-01
Hyposmia	Sore_Throat	Headache	1.09E-04	1.50E+01	4.44E-03	9.03E-02	7.93E-02
Sore_Throat	Dysgeusia	Fever	1.08E-04	1.50E+01	4.44E-03	7.36E-02	6.86E-02
Fatigue	Sore_Throat	Dysgeusia	1.66E-04	1.42E+01	5.29E-03	1.25E-01	1.02E-01
Sore_Throat	Cough	Dysgeusia	1.53E-04	1.43E+01	5.29E-03	1.05E-01	8.25E-02
Hyposmia	Sore_Throat	Cough	2.05E-04	1.38E+01	5.86E-03	8.28E-02	7.45E-02
Fatigue	Dysgeusia	Headache	2.47E-04	1.34E+01	6.43E-03	1.76E-01	1.36E-01
Cough	Dysgeusia	COVID_19_Contact	4.11E-04	1.25E+01	9.78E-03	7.28E-02	7.03E-02
Sore_Throat	Malaise	Dysgeusia	5.26E-04	1.20E+01	1.16E-02	1.27E-01	1.09E-01
Hyposmia	Dyspnea	Fever	5.71E-04	1.19E+01	1.17E-02	2.90E-02	1.94E-02
Sneezing	Diarrhea	Headache	6.57E-04	1.16E+01	1.25E-02	4.35E-02	2.54E-02
Cough	Malaise	Dysgeusia	7.95E-04	1.13E+01	1.32E-02	1.58E-01	1.36E-01
Hyposmia	Cough	Rhinorrhea	8.28E-04	1.12E+01	1.32E-02	7.43E-02	5.94E-02
Hyposmia	Dyspnea	Headache	8.13E-04	1.12E+01	1.32E-02	3.97E-02	2.49E-02
Malaise	Sneezing	Fever	1.06E-03	1.07E+01	1.59E-02	1.37E-01	1.33E-01
Fatigue	Hyposmia	Sore_Throat	1.22E-03	1.05E+01	1.75E-02	1.01E-01	9.19E-02
Hyposmia	Sore_Throat	Malaise	1.47E-03	1.01E+01	2.00E-02	1.03E-01	9.79E-02
Cough	Dysgeusia	Headache	1.71E-03	9.84E+00	2.04E-02	1.31E-01	1.10E-01
Dysgeusia	Sneezing	Fever	1.71E-03	9.84E+00	2.04E-02	5.35E-02	4.87E-02
Hyposmia	Rhinorrhea	Sneezing	1.71E-03	9.84E+00	2.04E-02	7.24E-02	3.37E-02
Fatigue	Malaise	Dysgeusia	2.27E-03	9.32E+00	2.60E-02	2.13E-01	1.68E-01
Dyspnea	Dysgeusia	Fever	2.74E-03	8.97E+00	2.90E-02	4.05E-02	2.15E-02
Dyspnea	Fever	Headache	2.73E-03	8.98E+00	2.90E-02	6.87E-02	4.75E-02
Hyposmia	Cough	COVID_19_Contact	3.04E-03	8.79E+00	3.10E-02	5.79E-02	6.34E-02
Malaise	Dysgeusia	Headache	4.35E-03	8.13E+00	4.11E-02	1.77E-01	1.45E-01
Diarrhea	Headache	COVID_19_Contact	4.38E-03	8.12E+00	4.11E-02	3.08E-02	3.05E-02
Fatigue	Hyposmia	Headache	4.46E-03	8.09E+00	4.11E-02	1.46E-01	1.22E-01
Sore_Throat	Rhinorrhea	Dysgeusia	4.74E-03	7.97E+00	4.24E-02	6.69E-02	5.26E-02
Rhinorrhea	Malaise	Dysgeusia	5.10E-03	7.84E+00	4.30E-02	1.02E-01	8.66E-02
Fatigue	Hyposmia	Cough	5.11E-03	7.84E+00	4.30E-02	1.26E-01	1.15E-01

Hyposmia	Fever	Headache	8.06E-03	7.02E+00	6.54E-02	8.21E-02	8.25E-02
Hyposmia	Sore_Throat	Rhinorrhea	8.23E-03	6.98E+00	6.54E-02	5.91E-02	4.75E-02
Hyposmia	Rhinorrhea	Fever	9.21E-03	6.78E+00	6.94E-02	4.61E-02	4.94E-02
Hyposmia	Fever	Diarrhea	9.02E-03	6.82E+00	6.94E-02	2.67E-02	1.53E-02
Rhinorrhea	Malaise	Sneezing	1.06E-02	6.53E+00	7.79E-02	1.95E-01	1.02E-01
Cough	Rhinorrhea	Dysgeusia	1.10E-02	6.47E+00	7.86E-02	8.14E-02	6.59E-02
Hyposmia	Cough	Fever	1.16E-02	6.37E+00	8.08E-02	6.95E-02	7.75E-02
Dysgeusia	Diarrhea	COVID_19_Contact	1.41E-02	6.02E+00	9.30E-02	1.71E-02	1.38E-02
Fatigue	Rhinorrhea	Dysgeusia	1.43E-02	6.00E+00	9.30E-02	1.03E-01	8.13E-02
Rhinorrhea	Dysgeusia	Fever	1.43E-02	6.00E+00	9.30E-02	5.16E-02	5.48E-02
Fatigue	Sneezing	Headache	1.74E-02	5.65E+00	1.11E-01	2.02E-01	1.59E-01
Hyposmia	Dysgeusia	Diarrhea	1.83E-02	5.56E+00	1.14E-01	3.94E-02	8.85E-03
Hyposmia	Diarrhea	COVID_19_Contact	1.90E-02	5.50E+00	1.16E-01	1.56E-02	1.25E-02
Dysgeusia	Fever	Headache	1.96E-02	5.45E+00	1.17E-01	1.03E-01	9.14E-02
Fatigue	Hyposmia	Dysgeusia	2.01E-02	5.41E+00	1.17E-01	1.65E-01	5.55E-02
Hyposmia	Malaise	Dysgeusia	2.07E-02	5.35E+00	1.17E-01	1.62E-01	5.91E-02
Sneezing	Fever	Diarrhea	2.08E-02	5.34E+00	1.17E-01	3.05E-02	1.99E-02
Rhinorrhea	Dysgeusia	Headache	2.19E-02	5.26E+00	1.20E-01	8.51E-02	7.01E-02
Sore_Throat	Rhinorrhea	Malaise	2.33E-02	5.15E+00	1.26E-01	1.73E-01	1.43E-01
Rhinorrhea	Dysgeusia	Sneezing	2.76E-02	4.85E+00	1.46E-01	7.73E-02	3.73E-02
Rhinorrhea	Malaise	Fever	2.88E-02	4.78E+00	1.50E-01	1.56E-01	1.49E-01
Hyposmia	Malaise	Sneezing	3.07E-02	4.67E+00	1.54E-01	9.62E-02	6.95E-02
Malaise	Dysgeusia	COVID_19_Contact	3.03E-02	4.69E+00	1.54E-01	8.84E-02	9.24E-02
Hyposmia	Sneezing	Headache	3.17E-02	4.61E+00	1.56E-01	7.76E-02	5.62E-02
Malaise	Dysgeusia	Sneezing	3.26E-02	4.57E+00	1.58E-01	1.06E-01	7.70E-02
Hyposmia	Dyspnea	COVID_19_Contact	3.34E-02	4.52E+00	1.59E-01	2.12E-02	1.59E-02
Hyposmia	Cough	Malaise	3.63E-02	4.38E+00	1.70E-01	1.27E-01	1.22E-01
Hyposmia	Malaise	Headache	3.99E-02	4.22E+00	1.84E-01	1.46E-01	1.30E-01
Fatigue	Hyposmia	Diarrhea	4.19E-02	4.14E+00	1.90E-01	4.42E-02	2.26E-02
Dysgeusia	Sneezing	Headache	4.52E-02	4.01E+00	2.02E-01	8.77E-02	6.23E-02
Malaise	Dysgeusia	Fever	4.64E-02	3.97E+00	2.04E-01	1.19E-01	1.13E-01
Sore_Throat	Dyspnea	Headache	5.08E-02	3.81E+00	2.11E-01	7.95E-02	4.57E-02
Fatigue	Cough	Rhinorrhea	4.88E-02	3.88E+00	2.11E-01	1.98E-01	1.68E-01
Dyspnea	Sneezing	Diarrhea	4.97E-02	3.85E+00	2.11E-01	1.71E-02	5.99E-03
Fatigue	Dysgeusia	Sneezing	5.01E-02	3.84E+00	2.11E-01	1.07E-01	7.23E-02
Sore_Throat	Cough	Headache	5.53E-02	3.67E+00	2.26E-01	2.58E-01	1.82E-01
Fatigue	Hyposmia	Rhinorrhea	5.61E-02	3.65E+00	2.26E-01	9.47E-02	7.34E-02
Dyspnea	Dysgeusia	COVID_19_Contact	5.79E-02	3.60E+00	2.28E-01	2.90E-02	1.76E-02
Fatigue	Cough	Diarrhea	5.81E-02	3.59E+00	2.28E-01	7.13E-02	5.20E-02
Rhinorrhea	Diarrhea	Headache	5.90E-02	3.56E+00	2.28E-01	4.23E-02	2.86E-02
Dyspnea	Dysgeusia	Headache	6.23E-02	3.47E+00	2.38E-01	5.31E-02	2.76E-02
Hyposmia	Cough	Headache	6.83E-02	3.32E+00	2.54E-01	1.01E-01	9.92E-02

Hyposmia	Dyspnea	Malaise	6.83E-02	3.32E+00	2.54E-01	4.46E-02	3.07E-02
Cough	Fever	Diarrhea	7.00E-02	3.28E+00	2.57E-01	5.05E-02	3.50E-02
Dysgeusia	Sneezing	Diarrhea	7.36E-02	3.20E+00	2.66E-01	2.97E-02	1.15E-02
Cough	Dysgeusia	Fever	7.53E-02	3.16E+00	2.69E-01	8.84E-02	8.59E-02
Rhinorrhea	Malaise	COVID_19_Contact	8.05E-02	3.05E+00	2.84E-01	1.31E-01	1.22E-01
Fatigue	Dyspnea	Sneezing	8.39E-02	2.99E+00	2.87E-01	6.05E-02	3.76E-02
Hyposmia	Malaise	Diarrhea	8.43E-02	2.98E+00	2.87E-01	4.38E-02	2.41E-02
Hyposmia	Malaise	Fever	8.39E-02	2.99E+00	2.87E-01	9.62E-02	1.02E-01
Hyposmia	Rhinorrhea	Diarrhea	8.52E-02	2.96E+00	2.87E-01	2.82E-02	1.17E-02
Fatigue	Cough	Sneezing	8.99E-02	2.88E+00	2.99E-01	1.92E-01	1.50E-01
Sneezing	Fever	Headache	9.98E-02	2.71E+00	3.16E-01	1.15E-01	1.07E-01
Cough	Headache	COVID_19_Contact	1.03E-01	2.66E+00	3.16E-01	1.71E-01	1.55E-01
Dyspnea	Fever	Diarrhea	9.70E-02	2.75E+00	3.16E-01	1.86E-02	8.79E-03
Fatigue	Hyposmia	Fever	1.01E-01	2.69E+00	3.16E-01	9.21E-02	9.57E-02
Sore_Throat	Rhinorrhea	Headache	9.81E-02	2.74E+00	3.16E-01	1.48E-01	1.16E-01
Dysgeusia	Fever	COVID_19_Contact	9.96E-02	2.71E+00	3.16E-01	4.38E-02	5.85E-02
Dyspnea	Diarrhea	COVID_19_Contact	1.03E-01	2.66E+00	3.16E-01	9.29E-03	7.19E-03
Hyposmia	Cough	Sneezing	1.05E-01	2.63E+00	3.19E-01	7.28E-02	5.28E-02
Fatigue	Diarrhea	COVID_19_Contact	1.07E-01	2.59E+00	3.23E-01	3.94E-02	3.54E-02
Cough	Dyspnea	Dysgeusia	1.09E-01	2.57E+00	3.25E-01	5.42E-02	2.59E-02
Malaise	Diarrhea	COVID_19_Contact	1.11E-01	2.54E+00	3.26E-01	4.05E-02	3.77E-02
Fatigue	Sneezing	Diarrhea	1.13E-01	2.51E+00	3.29E-01	4.79E-02	2.95E-02
Hyposmia	Rhinorrhea	Headache	1.16E-01	2.47E+00	3.36E-01	7.58E-02	6.33E-02
Sore_Throat	Fever	Diarrhea	1.20E-01	2.42E+00	3.40E-01	3.27E-02	2.80E-02
Sore_Throat	Cough	Rhinorrhea	1.20E-01	2.42E+00	3.40E-01	1.45E-01	1.09E-01
Fatigue	Dysgeusia	COVID_19_Contact	1.21E-01	2.40E+00	3.40E-01	9.03E-02	8.68E-02
Dyspnea	Malaise	Fever	1.24E-01	2.36E+00	3.42E-01	8.40E-02	5.87E-02
Dysgeusia	Diarrhea	Headache	1.24E-01	2.37E+00	3.42E-01	3.97E-02	2.16E-02
Fatigue	Malaise	Fever	1.26E-01	2.34E+00	3.43E-01	3.90E-01	2.89E-01
Dyspnea	Malaise	Diarrhea	1.31E-01	2.27E+00	3.52E-01	2.53E-02	1.39E-02
Fever	Diarrhea	Headache	1.32E-01	2.27E+00	3.52E-01	4.94E-02	3.73E-02
Cough	Malaise	Fever	1.37E-01	2.22E+00	3.58E-01	3.08E-01	2.34E-01
Fatigue	Headache	COVID_19_Contact	1.35E-01	2.23E+00	3.58E-01	2.17E-01	1.91E-01
Dysgeusia	Fever	Diarrhea	1.40E-01	2.17E+00	3.65E-01	2.82E-02	1.69E-02
Cough	Dyspnea	Rhinorrhea	1.43E-01	2.14E+00	3.69E-01	5.53E-02	3.42E-02
Fatigue	Dysgeusia	Fever	1.51E-01	2.06E+00	3.85E-01	1.14E-01	1.06E-01
Fatigue	Malaise	Headache	1.55E-01	2.02E+00	3.93E-01	4.95E-01	3.70E-01
Cough	Dyspnea	Sneezing	1.60E-01	1.97E+00	3.93E-01	5.83E-02	3.04E-02
Hyposmia	Sore_Throat	COVID_19_Contact	1.61E-01	1.97E+00	3.93E-01	4.61E-02	5.07E-02
Fatigue	Malaise	Sneezing	1.59E-01	1.98E+00	3.93E-01	2.45E-01	1.97E-01
Hyposmia	Rhinorrhea	Dysgeusia	1.57E-01	2.00E+00	3.93E-01	8.88E-02	2.87E-02
Malaise	Fever	Headache	1.64E-01	1.93E+00	3.98E-01	3.50E-01	2.49E-01

Hyposmia	Malaise	COVID_19_Contact	1.68E-01	1.90E+00	4.04E-01	7.28E-02	8.34E-02
Cough	Malaise	Headache	1.74E-01	1.85E+00	4.12E-01	3.76E-01	2.99E-01
Fatigue	Fever	Headache	1.74E-01	1.85E+00	4.12E-01	3.24E-01	2.34E-01
Fatigue	Cough	COVID_19_Contact	1.80E-01	1.80E+00	4.18E-01	1.97E-01	1.80E-01
Fatigue	Rhinorrhea	Sneezing	1.80E-01	1.80E+00	4.18E-01	1.85E-01	9.56E-02
Sore_Throat	Malaise	COVID_19_Contact	1.88E-01	1.73E+00	4.35E-01	1.76E-01	1.53E-01
Sore_Throat	Rhinorrhea	Fever	1.90E-01	1.72E+00	4.35E-01	9.88E-02	9.07E-02
Hyposmia	Dyspnea	Sneezing	1.94E-01	1.69E+00	4.40E-01	2.41E-02	1.33E-02
Dyspnea	Dysgeusia	Sneezing	1.98E-01	1.66E+00	4.43E-01	3.23E-02	1.47E-02
Hyposmia	Cough	Diarrhea	1.98E-01	1.66E+00	4.43E-01	3.38E-02	1.83E-02
Fatigue	Fever	Diarrhea	2.03E-01	1.62E+00	4.50E-01	5.61E-02	4.33E-02
Hyposmia	Sneezing	Diarrhea	2.08E-01	1.58E+00	4.59E-01	2.79E-02	1.04E-02
Rhinorrhea	Sneezing	Fever	2.14E-01	1.55E+00	4.67E-01	1.00E-01	6.44E-02
Fatigue	Hyposmia	COVID_19_Contact	2.17E-01	1.52E+00	4.70E-01	7.58E-02	7.83E-02
Sore_Throat	Dysgeusia	Diarrhea	2.24E-01	1.48E+00	4.82E-01	2.82E-02	1.62E-02
Fever	Diarrhea	COVID_19_Contact	2.28E-01	1.45E+00	4.86E-01	2.34E-02	2.38E-02
Sore_Throat	Dyspnea	Malaise	2.29E-01	1.45E+00	4.86E-01	8.73E-02	5.64E-02
Rhinorrhea	Sneezing	COVID_19_Contact	2.33E-01	1.42E+00	4.90E-01	1.04E-01	5.27E-02
Malaise	Sneezing	Diarrhea	2.45E-01	1.35E+00	5.12E-01	4.87E-02	3.14E-02
Cough	Dysgeusia	Sneezing	2.52E-01	1.31E+00	5.21E-01	8.28E-02	5.85E-02
Hyposmia	Dyspnea	Dysgeusia	2.61E-01	1.27E+00	5.36E-01	4.23E-02	1.13E-02
Dyspnea	Rhinorrhea	Fever	2.66E-01	1.24E+00	5.37E-01	3.83E-02	2.85E-02
Fatigue	Hyposmia	Malaise	2.66E-01	1.24E+00	5.37E-01	1.80E-01	1.51E-01
Dyspnea	Malaise	Dysgeusia	2.67E-01	1.23E+00	5.37E-01	6.13E-02	3.41E-02
Cough	Sneezing	Headache	2.71E-01	1.21E+00	5.39E-01	1.58E-01	1.29E-01
Dysgeusia	Headache	COVID_19_Contact	2.72E-01	1.21E+00	5.39E-01	7.32E-02	7.48E-02
Rhinorrhea	Diarrhea	COVID_19_Contact	2.74E-01	1.20E+00	5.40E-01	2.34E-02	1.83E-02
Hyposmia	Fever	COVID_19_Contact	2.76E-01	1.19E+00	5.41E-01	3.34E-02	5.27E-02
Malaise	Headache	COVID_19_Contact	2.86E-01	1.14E+00	5.57E-01	2.26E-01	2.04E-01
Fatigue	Sore_Throat	Diarrhea	2.91E-01	1.12E+00	5.58E-01	5.01E-02	4.16E-02
Cough	Rhinorrhea	Malaise	2.89E-01	1.12E+00	5.58E-01	2.09E-01	1.79E-01
Rhinorrhea	Dysgeusia	COVID_19_Contact	3.02E-01	1.07E+00	5.72E-01	4.94E-02	4.48E-02
Hyposmia	Cough	Dysgeusia	3.02E-01	1.06E+00	5.72E-01	1.17E-01	4.50E-02
Cough	Dyspnea	Diarrhea	3.25E-01	9.69E-01	5.82E-01	2.27E-02	1.06E-02
Sore_Throat	Rhinorrhea	COVID_19_Contact	3.12E-01	1.02E+00	5.82E-01	9.06E-02	7.43E-02
Hyposmia	Diarrhea	Headache	3.13E-01	1.02E+00	5.82E-01	3.53E-02	1.95E-02
Dyspnea	Sneezing	Fever	3.27E-01	9.60E-01	5.82E-01	3.90E-02	2.53E-02
Sneezing	Fever	COVID_19_Contact	3.19E-01	9.93E-01	5.82E-01	6.50E-02	6.87E-02
Sneezing	Diarrhea	COVID_19_Contact	3.27E-01	9.59E-01	5.82E-01	2.38E-02	1.63E-02
Rhinorrhea	Sneezing	Headache	3.21E-01	9.87E-01	5.82E-01	1.55E-01	8.24E-02
Rhinorrhea	Headache	COVID_19_Contact	3.21E-01	9.83E-01	5.82E-01	1.08E-01	9.89E-02
Cough	Fever	Headache	3.15E-01	1.01E+00	5.82E-01	2.54E-01	1.89E-01

Sore_Throat	Diarrhea	Headache	3.20E-01	9.90E-01	5.82E-01	4.53E-02	3.58E-02
Sore_Throat	Dysgeusia	COVID_19_Contact	3.35E-01	9.29E-01	5.88E-01	5.65E-02	5.62E-02
Fatigue	Sneezing	COVID_19_Contact	3.34E-01	9.34E-01	5.88E-01	1.24E-01	1.02E-01
Rhinorrhea	Malaise	Diarrhea	3.43E-01	9.00E-01	5.94E-01	4.94E-02	3.53E-02
Malaise	Sneezing	COVID_19_Contact	3.42E-01	9.05E-01	5.94E-01	1.30E-01	1.09E-01
Cough	Rhinorrhea	COVID_19_Contact	3.52E-01	8.68E-01	6.06E-01	1.07E-01	9.29E-02
Fatigue	Cough	Headache	3.62E-01	8.30E-01	6.11E-01	3.56E-01	2.81E-01
Sore_Throat	Cough	Malaise	3.58E-01	8.45E-01	6.11E-01	2.98E-01	2.25E-01
Dyspnea	Rhinorrhea	Diarrhea	3.60E-01	8.39E-01	6.11E-01	1.49E-02	6.74E-03
Sore_Throat	Dyspnea	Fever	3.63E-01	8.27E-01	6.11E-01	6.05E-02	3.57E-02
Fatigue	Sore_Throat	Dyspnea	3.68E-01	8.09E-01	6.16E-01	8.73E-02	5.30E-02
Cough	Rhinorrhea	Headache	3.74E-01	7.89E-01	6.23E-01	1.69E-01	1.45E-01
Fatigue	Dyspnea	Fever	3.77E-01	7.80E-01	6.23E-01	8.14E-02	5.51E-02
Cough	Sneezing	Fever	3.81E-01	7.66E-01	6.25E-01	1.08E-01	1.01E-01
Rhinorrhea	Fever	COVID_19_Contact	3.84E-01	7.59E-01	6.25E-01	6.87E-02	7.73E-02
Fatigue	Diarrhea	Headache	3.85E-01	7.56E-01	6.25E-01	7.39E-02	5.54E-02
Hyposmia	Sore_Throat	Dyspnea	3.88E-01	7.45E-01	6.27E-01	2.97E-02	1.87E-02
Cough	Dysgeusia	Diarrhea	3.91E-01	7.37E-01	6.28E-01	3.71E-02	2.03E-02
Hyposmia	Dysgeusia	Fever	3.94E-01	7.25E-01	6.30E-01	8.25E-02	3.74E-02
Rhinorrhea	Malaise	Headache	3.99E-01	7.11E-01	6.34E-01	2.18E-01	1.91E-01
Hyposmia	Cough	Dyspnea	4.12E-01	6.74E-01	6.51E-01	3.71E-02	2.34E-02
Cough	Rhinorrhea	Diarrhea	4.35E-01	6.11E-01	6.83E-01	4.09E-02	2.69E-02
Dyspnea	Dysgeusia	Diarrhea	4.44E-01	5.85E-01	6.94E-01	1.45E-02	5.10E-03
Sore_Throat	Cough	Fever	4.48E-01	5.76E-01	6.96E-01	1.91E-01	1.42E-01
Sore_Throat	Fever	COVID_19_Contact	4.52E-01	5.67E-01	6.98E-01	9.84E-02	9.68E-02
Fatigue	Sore_Throat	Headache	4.56E-01	5.56E-01	7.01E-01	3.02E-01	2.25E-01
Rhinorrhea	Dysgeusia	Diarrhea	4.74E-01	5.14E-01	7.24E-01	2.71E-02	1.30E-02
Cough	Dyspnea	COVID_19_Contact	4.80E-01	4.98E-01	7.27E-01	5.05E-02	3.65E-02
Dyspnea	Fever	COVID_19_Contact	4.81E-01	4.98E-01	7.27E-01	3.31E-02	3.04E-02
Sore_Throat	Cough	Sneezing	4.86E-01	4.85E-01	7.27E-01	1.42E-01	9.70E-02
Dyspnea	Malaise	Sneezing	4.93E-01	4.69E-01	7.27E-01	6.24E-02	4.00E-02
Sore_Throat	Headache	COVID_19_Contact	4.93E-01	4.70E-01	7.27E-01	1.48E-01	1.24E-01
Dyspnea	Sneezing	COVID_19_Contact	4.92E-01	4.72E-01	7.27E-01	3.31E-02	2.07E-02
Cough	Dyspnea	Headache	4.93E-01	4.70E-01	7.27E-01	9.14E-02	5.71E-02
Fatigue	Sneezing	Fever	4.96E-01	4.63E-01	7.28E-01	1.32E-01	1.25E-01
Hyposmia	Rhinorrhea	COVID_19_Contact	5.26E-01	4.02E-01	7.36E-01	4.53E-02	4.04E-02
Fatigue	Rhinorrhea	COVID_19_Contact	5.23E-01	4.07E-01	7.36E-01	1.23E-01	1.15E-01
Fatigue	Dyspnea	Malaise	5.12E-01	4.31E-01	7.36E-01	1.24E-01	8.72E-02
Hyposmia	Dysgeusia	COVID_19_Contact	5.26E-01	4.02E-01	7.36E-01	7.50E-02	3.06E-02
Dyspnea	Rhinorrhea	Sneezing	5.23E-01	4.07E-01	7.36E-01	4.79E-02	1.94E-02
Sore_Throat	Rhinorrhea	Diarrhea	5.25E-01	4.04E-01	7.36E-01	3.08E-02	2.15E-02
Sore_Throat	Sneezing	Headache	5.06E-01	4.43E-01	7.36E-01	1.40E-01	1.03E-01

Sore_Throat	Malaise	Sneezing	5.14E-01	4.27E-01	7.36E-01	1.67E-01	1.28E-01
Cough	Sneezing	Diarrhea	5.26E-01	4.02E-01	7.36E-01	3.83E-02	2.39E-02
Cough	Sneezing	COVID_19_Contact	5.28E-01	3.99E-01	7.36E-01	1.03E-01	8.26E-02
Sore_Throat	Cough	COVID_19_Contact	5.34E-01	3.86E-01	7.42E-01	1.42E-01	1.16E-01
Hyposmia	Dysgeusia	Sneezing	5.41E-01	3.74E-01	7.45E-01	9.14E-02	2.55E-02
Fatigue	Dysgeusia	Diarrhea	5.42E-01	3.72E-01	7.45E-01	4.61E-02	2.51E-02
Fatigue	Sore_Throat	Rhinorrhea	5.66E-01	3.29E-01	7.58E-01	1.59E-01	1.35E-01
Sore_Throat	Diarrhea	COVID_19_Contact	5.58E-01	3.43E-01	7.58E-01	2.75E-02	2.29E-02
Cough	Diarrhea	COVID_19_Contact	5.57E-01	3.46E-01	7.58E-01	3.53E-02	2.87E-02
Malaise	Sneezing	Headache	5.67E-01	3.27E-01	7.58E-01	2.06E-01	1.70E-01
Sore_Throat	Malaise	Diarrhea	5.67E-01	3.27E-01	7.58E-01	5.35E-02	4.43E-02
Sore_Throat	Rhinorrhea	Sneezing	5.65E-01	3.31E-01	7.58E-01	1.27E-01	6.19E-02
Sore_Throat	Malaise	Headache	5.95E-01	2.82E-01	7.80E-01	3.21E-01	2.39E-01
Sore_Throat	Dysgeusia	Sneezing	6.02E-01	2.72E-01	7.80E-01	6.76E-02	4.68E-02
Malaise	Dysgeusia	Diarrhea	5.98E-01	2.78E-01	7.80E-01	4.61E-02	2.67E-02
Fatigue	Sore_Throat	Sneezing	6.03E-01	2.71E-01	7.80E-01	1.58E-01	1.20E-01
Sore_Throat	Dyspnea	Rhinorrhea	5.87E-01	2.95E-01	7.80E-01	4.46E-02	2.74E-02
Fatigue	Hyposmia	Sneezing	6.01E-01	2.73E-01	7.80E-01	9.44E-02	6.52E-02
Dyspnea	Rhinorrhea	Malaise	5.95E-01	2.82E-01	7.80E-01	6.02E-02	4.50E-02
Fatigue	Hyposmia	Dyspnea	6.08E-01	2.63E-01	7.83E-01	4.38E-02	2.89E-02
Dyspnea	Rhinorrhea	Dysgeusia	6.12E-01	2.58E-01	7.85E-01	3.16E-02	1.65E-02
Fatigue	Sore_Throat	Cough	6.17E-01	2.50E-01	7.88E-01	2.76E-01	2.11E-01
Fatigue	Fever	COVID_19_Contact	6.24E-01	2.40E-01	7.90E-01	1.53E-01	1.50E-01
Cough	Malaise	Sneezing	6.24E-01	2.40E-01	7.90E-01	1.98E-01	1.60E-01
Sore_Throat	Dyspnea	Dysgeusia	6.36E-01	2.24E-01	8.00E-01	4.23E-02	2.07E-02
Fatigue	Rhinorrhea	Fever	6.38E-01	2.22E-01	8.00E-01	1.47E-01	1.40E-01
Hyposmia	Sore_Throat	Dysgeusia	6.45E-01	2.12E-01	8.06E-01	9.25E-02	3.59E-02
Sore_Throat	Malaise	Fever	6.53E-01	2.02E-01	8.12E-01	2.43E-01	1.87E-01
Rhinorrhea	Fever	Diarrhea	6.61E-01	1.93E-01	8.17E-01	2.93E-02	2.23E-02
Fatigue	Dyspnea	COVID_19_Contact	6.63E-01	1.90E-01	8.17E-01	5.50E-02	4.51E-02
Dyspnea	Rhinorrhea	Headache	6.80E-01	1.70E-01	8.34E-01	4.90E-02	3.65E-02
Sore_Throat	Sneezing	Fever	6.84E-01	1.65E-01	8.37E-01	9.18E-02	8.07E-02
Sore_Throat	Dyspnea	COVID_19_Contact	7.35E-01	1.14E-01	8.79E-01	4.20E-02	2.92E-02
Cough	Dyspnea	Fever	7.22E-01	1.26E-01	8.79E-01	7.28E-02	4.46E-02
Cough	Malaise	COVID_19_Contact	7.40E-01	1.10E-01	8.79E-01	2.06E-01	1.91E-01
Dyspnea	Rhinorrhea	COVID_19_Contact	7.40E-01	1.10E-01	8.79E-01	2.97E-02	2.33E-02
Sore_Throat	Fever	Headache	7.37E-01	1.13E-01	8.79E-01	2.11E-01	1.51E-01
Fatigue	Cough	Fever	7.39E-01	1.11E-01	8.79E-01	2.83E-01	2.20E-01
Fatigue	Malaise	Diarrhea	7.26E-01	1.23E-01	8.79E-01	8.88E-02	6.84E-02
Hyposmia	Sneezing	COVID_19_Contact	7.46E-01	1.05E-01	8.82E-01	4.68E-02	3.60E-02
Sneezing	Headache	COVID_19_Contact	7.64E-01	9.04E-02	8.90E-01	1.05E-01	8.79E-02
Rhinorrhea	Fever	Headache	7.69E-01	8.64E-02	8.90E-01	1.32E-01	1.21E-01

Cough	Malaise	Diarrhea	7.72E-01	8.43E-02	8.90E-01	7.28E-02	5.54E-02
Cough	Fever	COVID_19_Contact	7.69E-01	8.62E-02	8.90E-01	1.23E-01	1.21E-01
Sore_Throat	Dyspnea	Sneezing	7.64E-01	9.03E-02	8.90E-01	4.79E-02	2.43E-02
Rhinorrhea	Sneezing	Diarrhea	7.69E-01	8.62E-02	8.90E-01	3.90E-02	1.52E-02
Dyspnea	Malaise	COVID_19_Contact	7.81E-01	7.73E-02	8.93E-01	5.53E-02	4.80E-02
Fatigue	Cough	Malaise	7.81E-01	7.73E-02	8.93E-01	4.38E-01	3.47E-01
Hyposmia	Dyspnea	Rhinorrhea	7.87E-01	7.30E-02	8.93E-01	2.41E-02	1.49E-02
Malaise	Diarrhea	Headache	7.87E-01	7.32E-02	8.93E-01	7.58E-02	5.90E-02
Cough	Rhinorrhea	Sneezing	7.97E-01	6.62E-02	9.01E-01	1.54E-01	7.74E-02
Hyposmia	Sore_Throat	Sneezing	8.13E-01	5.60E-02	9.15E-01	5.76E-02	4.22E-02
Fatigue	Rhinorrhea	Diarrhea	8.22E-01	5.09E-02	9.18E-01	4.64E-02	3.32E-02
Fatigue	Dyspnea	Diarrhea	8.20E-01	5.19E-02	9.18E-01	2.38E-02	1.31E-02
Dyspnea	Sneezing	Headache	8.31E-01	4.57E-02	9.25E-01	5.27E-02	3.24E-02
Cough	Diarrhea	Headache	8.37E-01	4.23E-02	9.28E-01	5.91E-02	4.48E-02
Fatigue	Rhinorrhea	Headache	8.40E-01	4.07E-02	9.28E-01	2.08E-01	1.79E-01
Fatigue	Sore_Throat	Malaise	8.43E-01	3.90E-02	9.28E-01	3.53E-01	2.78E-01
Fatigue	Dyspnea	Rhinorrhea	8.56E-01	3.29E-02	9.33E-01	5.91E-02	4.23E-02
Hyposmia	Headache	COVID_19_Contact	8.64E-01	2.91E-02	9.33E-01	5.79E-02	6.75E-02
Fatigue	Sore_Throat	Fever	8.59E-01	3.14E-02	9.33E-01	2.23E-01	1.76E-01
Dyspnea	Malaise	Headache	8.60E-01	3.12E-02	9.33E-01	1.05E-01	7.51E-02
Hyposmia	Sore_Throat	Diarrhea	8.63E-01	2.99E-02	9.33E-01	2.34E-02	1.47E-02
Sore_Throat	Cough	Dyspnea	8.73E-01	2.56E-02	9.39E-01	7.95E-02	4.29E-02
Cough	Dyspnea	Malaise	8.79E-01	2.32E-02	9.42E-01	1.09E-01	7.06E-02
Sore_Throat	Sneezing	COVID_19_Contact	8.86E-01	2.05E-02	9.42E-01	9.03E-02	6.60E-02
Fatigue	Dyspnea	Headache	8.84E-01	2.15E-02	9.42E-01	1.03E-01	7.06E-02
Cough	Rhinorrhea	Fever	8.90E-01	1.90E-02	9.43E-01	1.21E-01	1.13E-01
Sore_Throat	Dyspnea	Diarrhea	9.10E-01	1.27E-02	9.61E-01	1.67E-02	8.45E-03
Sore_Throat	Cough	Diarrhea	9.33E-01	7.04E-03	9.81E-01	4.57E-02	3.37E-02
Hyposmia	Dyspnea	Diarrhea	9.41E-01	5.50E-03	9.86E-01	1.23E-02	4.60E-03
Fatigue	Rhinorrhea	Malaise	9.51E-01	3.74E-03	9.86E-01	2.57E-01	2.22E-01
Fatigue	Sore_Throat	COVID_19_Contact	9.50E-01	3.91E-03	9.86E-01	1.63E-01	1.44E-01
Fatigue	Malaise	COVID_19_Contact	9.48E-01	4.22E-03	9.86E-01	2.64E-01	2.36E-01
Dyspnea	Diarrhea	Headache	9.65E-01	1.95E-03	9.87E-01	2.04E-02	1.13E-02
Hyposmia	Dysgeusia	Headache	9.58E-01	2.81E-03	9.87E-01	1.33E-01	4.79E-02
Fatigue	Dyspnea	Dysgeusia	9.66E-01	1.82E-03	9.87E-01	6.05E-02	3.20E-02
Fever	Headache	COVID_19_Contact	9.61E-01	2.35E-03	9.87E-01	1.37E-01	1.29E-01
Fatigue	Cough	Dyspnea	9.70E-01	1.39E-03	9.88E-01	1.06E-01	6.63E-02
Dyspnea	Headache	COVID_19_Contact	9.78E-01	7.41E-04	9.92E-01	4.64E-02	3.89E-02
Malaise	Fever	COVID_19_Contact	9.87E-01	2.83E-04	9.96E-01	1.65E-01	1.59E-01
Malaise	Fever	Diarrhea	9.93E-01	8.56E-05	9.96E-01	6.13E-02	4.61E-02
Sore_Throat	Sneezing	Diarrhea	9.90E-01	1.70E-04	9.96E-01	3.05E-02	1.91E-02
Dysgeusia	Sneezing	COVID_19_Contact	9.96E-01	2.15E-05	9.96E-01	5.13E-02	3.99E-02

Supplementary Table 3: Coefficients of the Logistic Regression without interaction terms when trained on the full dataset (N=8966), including the Contact with a COVID-19 positively tested person information

	Coefficient (2.5%-97.5%)	P value
Intercept	-2.41 (-2.63,-2.19)	1.23E-102
COVID-19 contact	1.22 (1.11, 1.33)	4.26E-102
Cough	0.83 (0.73, 0.94)	2.25E-55
Hyposmia	1.01 (0.85, 1.18)	2.47E-33
Fever	0.53 (0.42, 0.64)	4.33E-22
Diarrhea	-0.58 (-0.73, -0.44)	1.33E-14
age	0.15 (0.10, 0.20)	2.40E-10
Sore Throat	-0.31 (-0.41, -0.21)	3.76E-09
Dyspnea	-0.42 (-0.57, -0.28)	5.91E-09
Sneezing	-0.34 (-0.46, -0.22)	2.37E-08
Headache	0.30 (0.19, 0.41)	4.86E-08
Dysgeusia	0.39 (0.24, 0.55)	8.41E-07
sex	-0.14 (-0.24, -0.04)	6.91E-03
Malaise	0.19 (0.05, 0.33)	7.03E-03
Rhinorrhea	-0.07 (-0.18, 0.05)	2.39E-01
Fatigue	-0.06 (-0.19, 0.06)	3.38E-01

Supplementary Table 4: Coefficients of the model including interaction terms

	Coefficient	2.5 %	97.5 %	P value
(Intercept)	-1.82E+00	-2.41E+00	-1.25E+00	9.08E-10
sex	-1.74E-01	-6.23E-01	2.74E-01	4.47E-01
age	1.47E-01	8.89E-03	2.86E-01	3.75E-02
Fatigue	-5.25E-02	-6.14E-01	5.08E-01	8.55E-01
Hyposmia	1.80E+00	9.97E-01	2.60E+00	1.13E-05
Sore_Throat	-1.31E-01	-6.14E-01	3.49E-01	5.94E-01
Cough	3.07E-02	-4.38E-01	4.99E-01	8.98E-01
Dyspnea	-1.54E+00	-2.33E+00	-7.72E-01	1.07E-04
Rhinorrhea	2.35E-01	-2.55E-01	7.23E-01	3.46E-01
Malaise	-6.74E-01	-1.29E+00	-6.50E-02	3.04E-02
Dysgeusia	1.12E+00	3.39E-01	1.89E+00	4.53E-03
Sneezing	-1.44E-01	-7.08E-01	4.14E-01	6.14E-01
Fever	-2.72E-01	-7.90E-01	2.41E-01	3.01E-01
Diarrhea	-8.69E-01	-1.64E+00	-1.24E-01	2.48E-02
Headache	9.07E-01	3.97E-01	1.42E+00	4.91E-04
COVID_19_Contact	3.08E-01	-1.52E-01	7.69E-01	1.90E-01
sex:age	-2.67E-02	-1.24E-01	7.10E-02	5.92E-01
sex:Fatigue	-1.73E-01	-4.37E-01	9.01E-02	1.97E-01
sex:Hyposmia	-2.38E-01	-5.87E-01	1.11E-01	1.81E-01
sex:Sore_Throat	-3.61E-02	-2.54E-01	1.82E-01	7.45E-01
sex:Cough	5.48E-02	-1.66E-01	2.75E-01	6.26E-01
sex:Dyspnea	3.00E-01	-2.37E-03	6.03E-01	5.23E-02
sex:Rhinorrhea	1.51E-01	-8.94E-02	3.91E-01	2.19E-01
sex:Malaise	1.08E-01	-1.83E-01	3.99E-01	4.66E-01
sex:Dysgeusia	-8.79E-02	-4.18E-01	2.43E-01	6.02E-01
sex:Sneezing	1.78E-01	-7.40E-02	4.31E-01	1.66E-01
sex:Fever	4.94E-02	-1.78E-01	2.77E-01	6.70E-01

sex:Diarrhea	5.67E-02	-2.72E-01	3.85E-01	7.35E-01
sex:Headache	-4.59E-02	-2.77E-01	1.85E-01	6.97E-01
sex:COVID_19_Contact	1.56E-01	-8.01E-02	3.93E-01	1.95E-01
age:Fatigue	-3.05E-02	-1.51E-01	8.94E-02	6.18E-01
age:Hyposmia	1.55E-02	-1.52E-01	1.83E-01	8.56E-01
age:Sore_Throat	-1.24E-01	-2.28E-01	-1.89E-02	2.06E-02
age:Cough	1.33E-01	3.11E-02	2.35E-01	1.05E-02
age:Dyspnea	1.44E-01	-1.18E-03	2.90E-01	5.28E-02
age:Rhinorrhea	-1.27E-02	-1.20E-01	9.44E-02	8.17E-01
age:Malaise	7.51E-02	-5.19E-02	2.02E-01	2.46E-01
age:Dysgeusia	-9.53E-02	-2.54E-01	6.41E-02	2.40E-01
age:Sneezing	-3.33E-02	-1.46E-01	8.01E-02	5.64E-01
age:Fever	2.34E-01	1.28E-01	3.40E-01	1.45E-05
age:Diarrhea	-4.66E-02	-2.02E-01	1.11E-01	5.59E-01
age:Headache	-2.14E-01	-3.21E-01	-1.07E-01	9.39E-05
age:COVID_19_Contact	-3.68E-02	-1.40E-01	6.64E-02	4.85E-01
Fatigue:Hyposmia	3.54E-01	-1.41E-01	8.49E-01	1.61E-01
Fatigue:Sore_Throat	1.05E-01	-1.68E-01	3.79E-01	4.51E-01
Fatigue:Cough	1.75E-01	-9.64E-02	4.46E-01	2.05E-01
Fatigue:Dyspnea	4.58E-02	-3.96E-01	4.97E-01	8.40E-01
Fatigue:Rhinorrhea	2.61E-01	-4.26E-02	5.65E-01	9.22E-02
Fatigue:Malaise	6.10E-02	-2.38E-01	3.61E-01	6.90E-01
Fatigue:Dysgeusia	-5.22E-02	-5.38E-01	4.36E-01	8.33E-01
Fatigue:Sneezing	-1.14E-01	-4.40E-01	2.13E-01	4.96E-01
Fatigue:Fever	-5.00E-02	-3.39E-01	2.38E-01	7.34E-01
Fatigue:Diarrhea	-5.04E-01	-9.41E-01	-6.22E-02	2.44E-02
Fatigue:Headache	-9.27E-02	-3.62E-01	1.76E-01	5.00E-01
Fatigue:COVID_19_Contact	4.09E-02	-2.55E-01	3.36E-01	7.86E-01
Hyposmia:Sore_Throat	-2.89E-01	-6.56E-01	7.68E-02	1.22E-01
Hyposmia:Cough	-5.10E-01	-8.85E-01	-1.35E-01	7.73E-03

Hyposmia:Dyspnea	-7.07E-01	-1.14E+00	-2.74E-01	1.42E-03
Hyposmia:Rhinorrhea	9.91E-02	-2.84E-01	4.83E-01	6.13E-01
Hyposmia:Malaise	-1.96E-01	-7.11E-01	3.14E-01	4.53E-01
Hyposmia:Dysgeusia	4.12E-03	-3.48E-01	3.58E-01	9.82E-01
Hyposmia:Sneezing	-2.67E-01	-6.53E-01	1.18E-01	1.74E-01
Hyposmia:Fever	-5.56E-01	-9.26E-01	-1.86E-01	3.22E-03
Hyposmia:Diarrhea	6.19E-01	1.67E-01	1.08E+00	7.51E-03
Hyposmia:Headache	-4.55E-02	-4.39E-01	3.48E-01	8.20E-01
Hyposmia:COVID_19_Contact	-2.40E-01	-6.29E-01	1.53E-01	2.30E-01
Sore_Throat:Cough	-1.31E-01	-3.54E-01	9.16E-02	2.48E-01
Sore_Throat:Dyspnea	5.18E-01	2.04E-01	8.32E-01	1.24E-03
Sore_Throat:Rhinorrhea	-2.81E-01	-5.34E-01	-2.87E-02	2.93E-02
Sore_Throat:Malaise	-3.17E-02	-3.33E-01	2.70E-01	8.37E-01
Sore_Throat:Dysgeusia	-1.11E-01	-4.56E-01	2.33E-01	5.26E-01
Sore_Throat:Sneezing	5.37E-02	-2.11E-01	3.18E-01	6.91E-01
Sore_Throat:Fever	2.38E-01	1.17E-03	4.74E-01	4.88E-02
Sore_Throat:Diarrhea	2.00E-01	-1.44E-01	5.41E-01	2.53E-01
Sore_Throat:Headache	1.40E-01	-9.52E-02	3.75E-01	2.44E-01
Sore_Throat:COVID_19_Contact	5.27E-01	2.85E-01	7.68E-01	1.87E-05
Cough:Dyspnea	-2.42E-01	-5.69E-01	9.16E-02	1.51E-01
Cough:Rhinorrhea	-5.68E-01	-8.18E-01	-3.17E-01	8.80E-06
Cough:Malaise	5.69E-01	2.70E-01	8.69E-01	1.94E-04
Cough:Dysgeusia	-1.30E-01	-4.83E-01	2.27E-01	4.73E-01
Cough:Sneezing	-3.02E-01	-5.65E-01	-3.91E-02	2.42E-02
Cough:Fever	2.91E-01	5.28E-02	5.29E-01	1.66E-02
Cough:Diarrhea	3.48E-01	7.57E-03	6.93E-01	4.63E-02
Cough:Headache	1.80E-01	-6.03E-02	4.18E-01	1.41E-01
Cough:COVID_19_Contact	5.81E-02	-1.85E-01	3.02E-01	6.40E-01
Dyspnea:Rhinorrhea	9.82E-02	-2.45E-01	4.39E-01	5.73E-01

Dyspnea:Malaise	3.66E-02	-4.35E-01	5.20E-01	8.80E-01
Dyspnea:Dysgeusia	3.56E-01	-4.32E-02	7.56E-01	8.01E-02
Dyspnea:Sneezing	4.01E-01	5.56E-02	7.46E-01	2.27E-02
Dyspnea:Fever	-2.58E-03	-3.12E-01	3.08E-01	9.87E-01
Dyspnea:Diarrhea	1.87E-01	-2.37E-01	6.02E-01	3.81E-01
Dyspnea:Headache	9.53E-03	-3.30E-01	3.52E-01	9.56E-01
Dyspnea:COVID_19_Contact	1.47E-02	-3.17E-01	3.47E-01	9.31E-01
Rhinorrhea:Malaise	-2.15E-01	-5.44E-01	1.13E-01	1.98E-01
Rhinorrhea:Dysgeusia	-5.05E-01	-8.75E-01	-1.37E-01	7.33E-03
Rhinorrhea:Sneezing	2.74E-02	-2.24E-01	2.80E-01	8.31E-01
Rhinorrhea:Fever	-9.48E-02	-3.54E-01	1.65E-01	4.74E-01
Rhinorrhea:Diarrhea	3.93E-01	3.34E-02	7.51E-01	3.19E-02
Rhinorrhea:Headache	1.89E-01	-7.70E-02	4.56E-01	1.64E-01
Rhinorrhea:COVID_19_Contact	1.41E-01	-1.22E-01	4.05E-01	2.93E-01
Malaise:Dysgeusia	2.48E-02	-4.77E-01	5.34E-01	9.23E-01
Malaise:Sneezing	1.48E-01	-2.01E-01	4.98E-01	4.07E-01
Malaise:Fever	-3.12E-01	-6.48E-01	2.49E-02	6.89E-02
Malaise:Diarrhea	1.74E-01	-2.99E-01	6.59E-01	4.75E-01
Malaise:Headache	9.84E-02	-1.99E-01	3.96E-01	5.16E-01
Malaise:COVID_19_Contact	9.11E-01	5.98E-01	1.22E+00	1.14E-08
Dysgeusia:Sneezing	2.58E-01	-1.14E-01	6.29E-01	1.74E-01
Dysgeusia:Fever	-3.15E-01	-6.61E-01	3.17E-02	7.46E-02
Dysgeusia:Diarrhea	2.84E-02	-4.12E-01	4.63E-01	8.99E-01
Dysgeusia:Headache	5.45E-02	-3.18E-01	4.30E-01	7.75E-01
Dysgeusia:COVID_19_Contact	-3.92E-01	-7.58E-01	-2.54E-02	3.59E-02
Sneezing:Fever	-3.17E-02	-3.02E-01	2.38E-01	8.18E-01
Sneezing:Diarrhea	-5.77E-02	-4.26E-01	3.07E-01	7.58E-01
Sneezing:Headache	-1.54E-01	-4.35E-01	1.27E-01	2.83E-01
Sneezing:COVID_19_Contact	-5.33E-02	-3.27E-01	2.20E-01	7.03E-01

Fever:Diarrhea	-5.68E-02	-3.96E-01	2.84E-01	7.43E-01
Fever:Headache	1.61E-01	-8.53E-02	4.08E-01	2.00E-01
Fever:COVID_19_Contact	4.84E-01	2.28E-01	7.42E-01	2.25E-04
Diarrhea:Headache	1.71E-02	-3.49E-01	3.87E-01	9.28E-01
Diarrhea:COVID_19_Contact	1.95E-01	-1.58E-01	5.51E-01	2.81E-01
Headache:COVID_19_Contact	-2.18E-01	-4.75E-01	3.80E-02	9.56E-02

Supplementary Figure 1

A) C19+ group

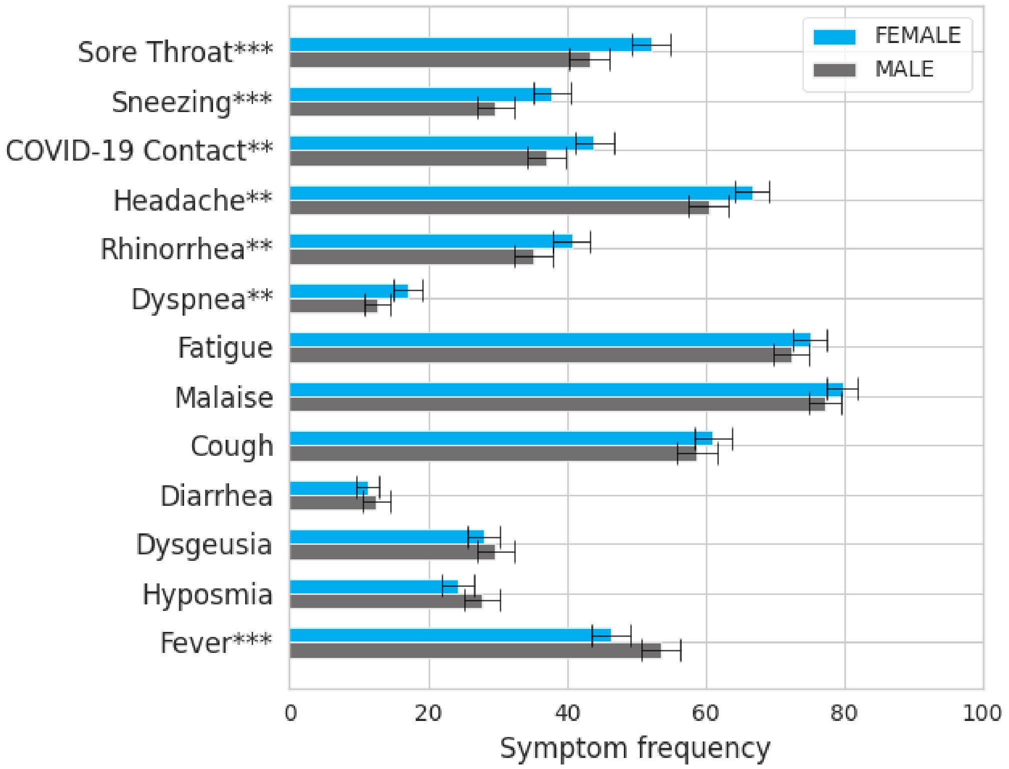

B) C19- group

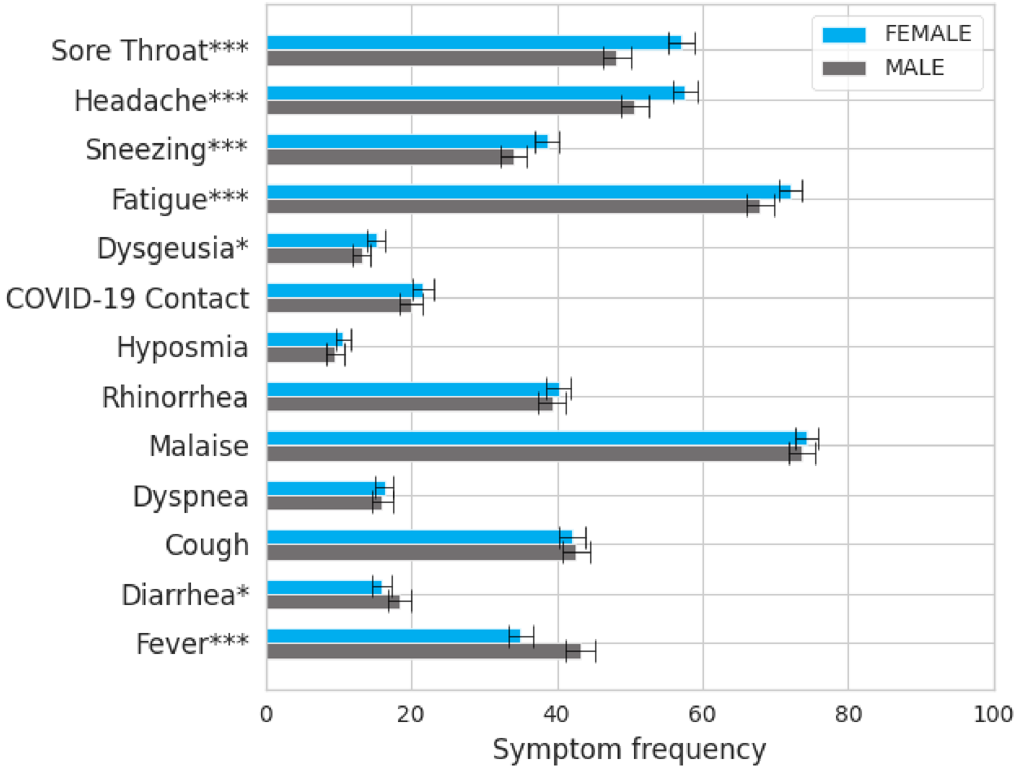

Supplementary Figure 1. Symptom frequencies among the female and male for the C19+ group and (B) for the C19- group. Error bars show the 95% confidence intervals. Symptoms with a p-value less than 0.05, 0.01, and 0.001 are indicated with one, two, and three asterisks respectively.

Supplementary Figure 2

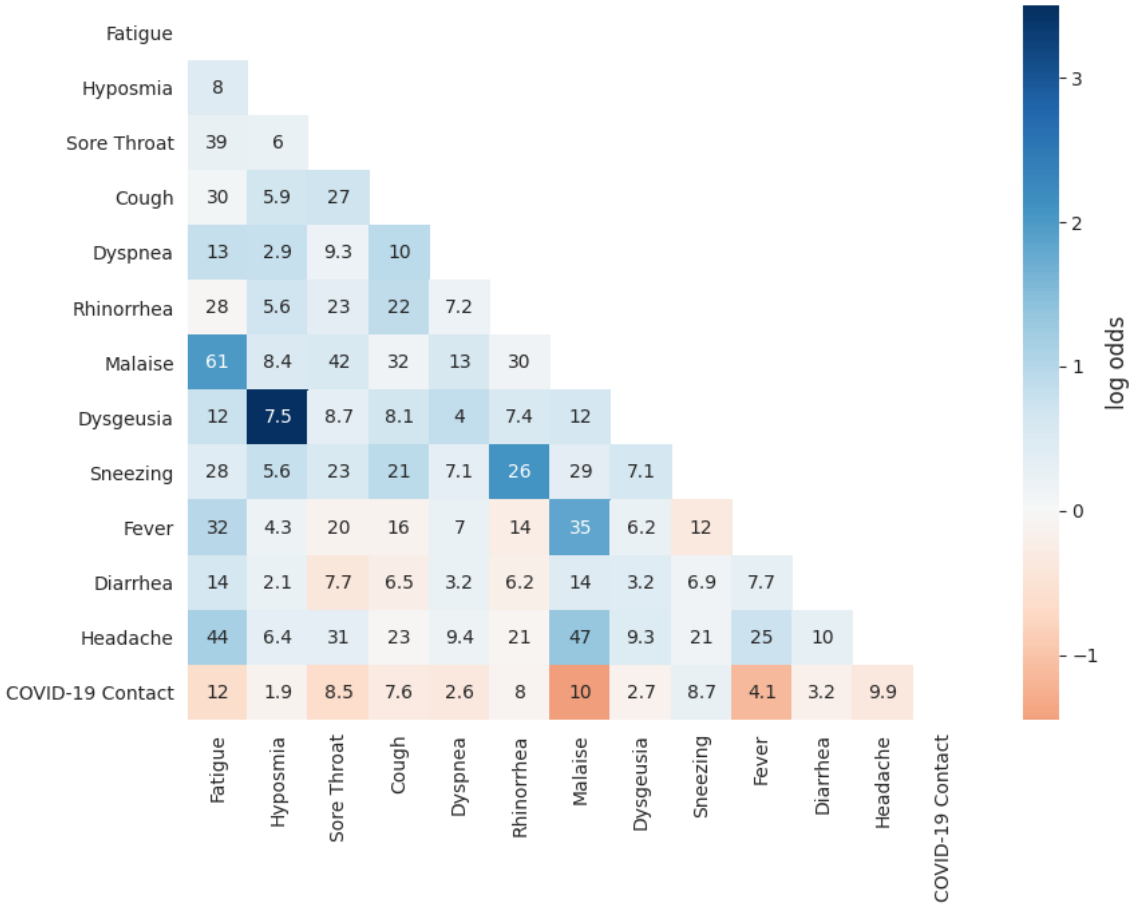

Supplementary Figure 2. Symptoms co-occurrence frequencies for the C19- group. Frequencies are reported in percentage. Log Odds Ratios (LOR) are represented by the color scale.

Supplementary Figure 3

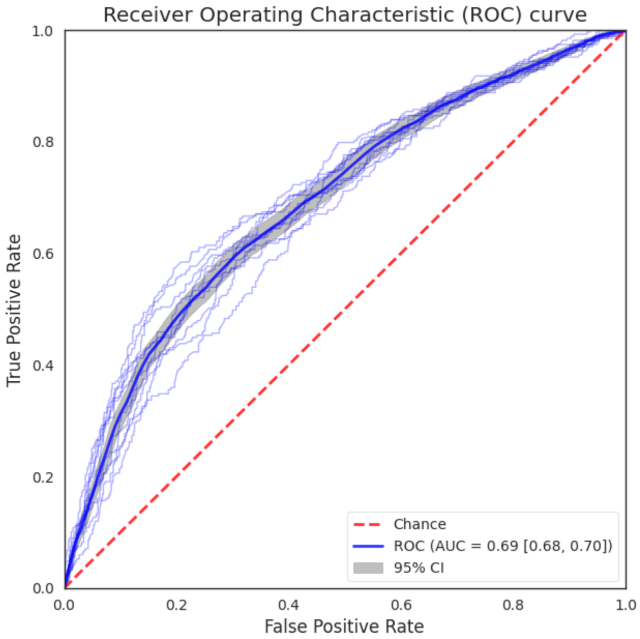

Supplementary Figure 3. Receiver operating characteristic (ROC) curve of the predictive model when not accounting for the contact with COVID-19 case information. Transparent band shows the 95% Confidence Intervals (CI). The area under the curve (AUC) is provided to summarise the curve.

Supplementary Figure 4

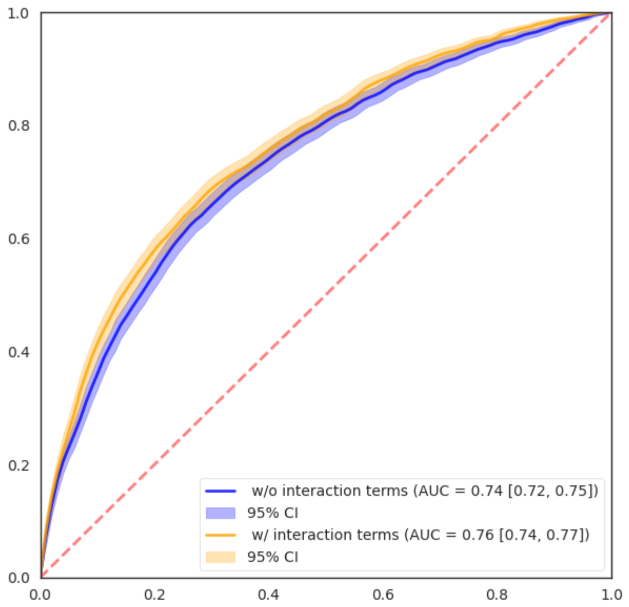

Supplementary Figure 4. Receiver operating characteristic (ROC) curve of the predictive model with the interaction terms in orange and without the interaction terms in blue. Transparent band shows the 95% Confidence Intervals (CI). The area under the curve (AUC) is provided to summarise the curve.

Supplementary Figure 5

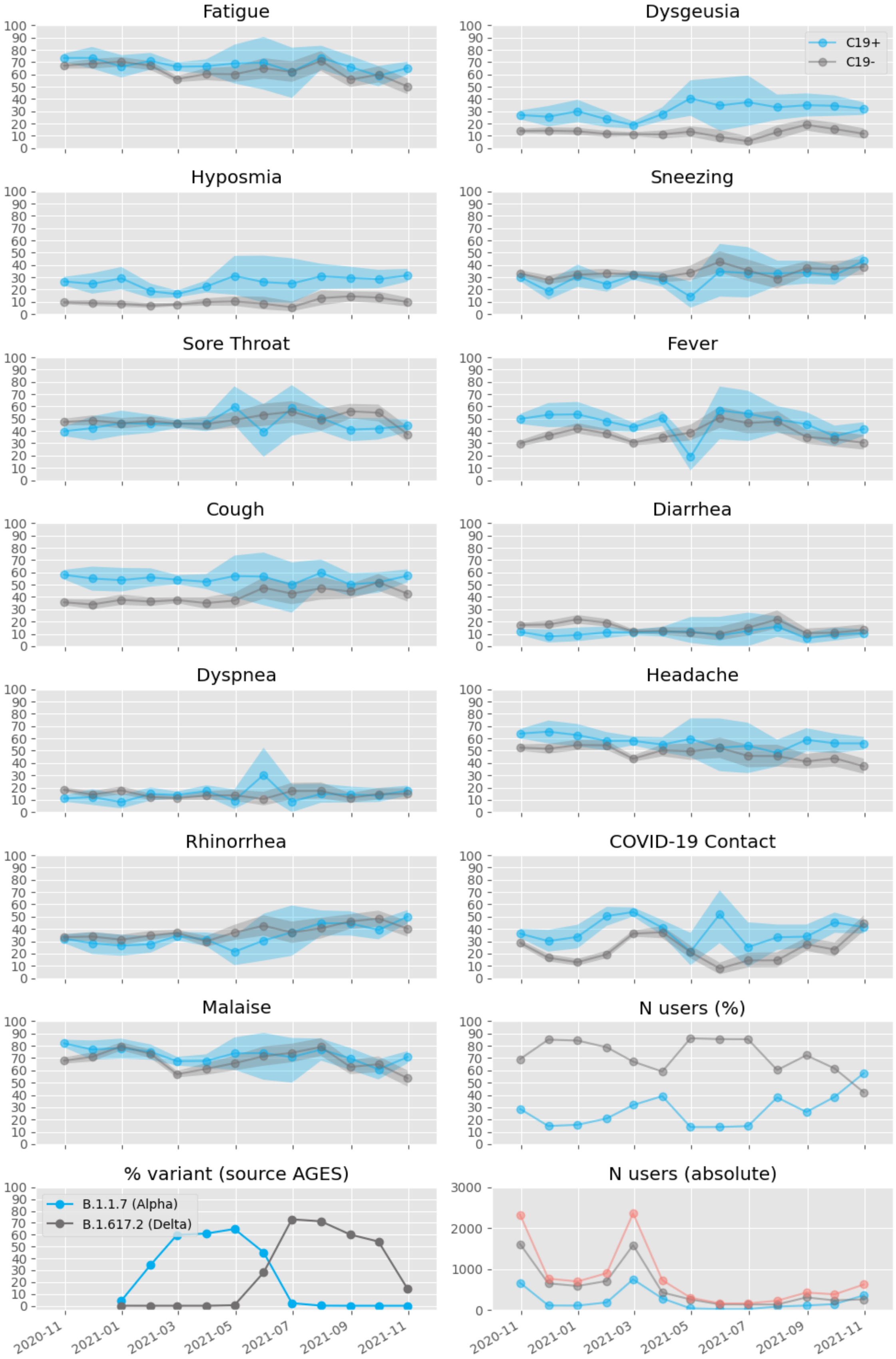

Supplementary Figure 5. Evolution of the symptom frequencies, the percentage of the Alpha and Delta variants detected in Austria and the number of users.
